# A Gene's Ability to Buffer Variation Is Predicted by Its Fitness Contribution and Genetic Interactions

**DOI:** 10.1371/journal.pone.0017650

**Published:** 2011-03-02

**Authors:** Guang-Zhong Wang, Jian Liu, Wei Wang, Hong-Yu Zhang, Martin J. Lercher

**Affiliations:** 1 Institute for Computer Science, Heinrich-Heine-University, Düsseldorf, Germany; 2 Shandong Provincial Research Center for Bioinformatic Engineering and Technique, Center for Advanced Study, Shandong University of Technology, Zibo, P. R. China; 3 Institute of Bioinformatics and State Key Laboratory of Crop Genetic Improvement, College of Life Science and Technology, Huazhong Agricultural University, Wuhan, P. R. China; Tulane University Health Sciences Center, United States of America

## Abstract

**Background:**

Many single-gene knockouts result in increased phenotypic (e.g., morphological) variability among the mutant's offspring. This has been interpreted as an intrinsic ability of genes to buffer genetic and environmental variation. A phenotypic capacitor is a gene that appears to mask phenotypic variation: when knocked out, the offspring shows more variability than the wild type. Theory predicts that this phenotypic potential should be correlated with a gene's knockout fitness and its number of negative genetic interactions. Based on experimentally measured phenotypic capacity, it was suggested that knockout fitness was unimportant, but that phenotypic capacitors tend to be hubs in genetic and physical interaction networks.

**Methodology/Principal Findings:**

We re-analyse the available experimental data in a combined model, which includes knockout fitness and network parameters as well as expression level and protein length as predictors of phenotypic potential. Contrary to previous conclusions, we find that the strongest predictor is in fact haploid knockout fitness (responsible for 9% of the variation in phenotypic potential), with an additional contribution from the genetic interaction network (5%); once these two factors are taken into account, protein-protein interactions do not make any additional contribution to the variation in phenotypic potential.

**Conclusions/Significance:**

We conclude that phenotypic potential is not a mysterious “emergent” property of cellular networks. Instead, it is very simply determined by the overall fitness reduction of the organism (which in its compromised state can no longer compensate for multiple factors that contribute to phenotypic variation), and by the number (and presumably nature) of genetic interactions of the knocked-out gene. In this light, Hsp90, the prototypical phenotypic capacitor, may not be representative: typical phenotypic capacitors are not direct “buffers” of variation, but are simply genes encoding central cellular functions.

## Introduction

One of the most fundamental and challenging problems in biology is the relationship between genotype and phenotype. Little is known about this relationship in most biological systems. The genotype-phenotype relationship is complicated by the fact that loss-of-function mutations of some individual genes are capable of increasing phenotypic variability in a wide range of traits; such genes are termed ‘phenotypic capacitors’ [1], [2], [3], [4], [5], [6], [7]. The induced variability may either be due to cryptic genetic variation that is released by the mutation [8], or may be non-genetic [9]. Phenotypic capacitors have been discovered in diverse species including *Drosophila*, *Arabidopsis*, *Manduca*, *Escherichia coli*, and yeast [4], [5], [6], [7], [10], [11], [12].

Theoretical simulations of complex cellular networks have suggested that most genes reveal cryptic genetic variation when functionally compromised [1]. More generally, phenotypic release of hidden genetic variation appears to be a generic property of models with epistasis or genotype-environment interactions [8]. This process may even be selectively favourable: an allele for the revelation of cryptic genetic variation can invade a population if revelation is sometimes selectively favourable [13]. However, it is unclear if such alleles do in fact exist, as phenotypic capacitance can arise as a direct consequence of network structure [1], [8].

The standard model of a phenotypic capacitor is the heat shock protein Hsp90, a chaperone that helps many proteins to achieve their correct 3-D structure. This assistance in folding likely removes some selective constraints on amino acid sequence evolution, as reported for another chaperone, GroEL [14], [15]. This direct buffering effect allows the accumulation of polymorphisms that would impede correct protein folding in the absence of the chaperone. Knockout of Hsp90 then releases these hidden polymorphisms, resulting in variation in protein folding efficiency between genetically different individuals [16]. However, the same study reported that – contrary to theoretical expectations – purely non-genetic phenotypic variation was not released by knocking out Hsp90 in flies [16].

Theory predicts that (i) phenotypic capacitors should have similar effects on genetically and non-genetically caused phenotypic variation [16]; (ii) for the majority of genes, strong functional impairment leads to the revelation of cryptic genetic variation [1], [8]; and (iii) the release of cryptic genetic variation by knockout mutations is related to genetic interactions of the mutated gene [8]. This suggests that phenotypic capacitors that reveal genetically caused phenotypic variation should often be genes with severe knockout-effects and with many synthetic lethal interactions, and that the same may be true for non-genetically caused phenotypic variation.

In a recent study, Levy and Siegal examined non-genetic variation among yeast cells [9]. They found more than 300 phenotypic capacitors, and reported that many of these had a large number of synthetic lethal interactions; at the same time, the authors reported that they did not observe strong effects of the knockouts on growth rates. These findings were interpreted as evidence for incomplete functional redundancy at multiple levels in the genetic architecture [9]. However, Levy and Siegal may have underestimated the biological significance of the ‘weak’ fitness effects of knockouts. Consequently, they failed to directly compare the effects of network parameters and knockout fitness in a combined model.

Here, we re-examine this issue, employing a combined general linear model to test genetic interactions and knockout fitness effects as well as three additional variables that might affect ‘phenotypic potential’, *i.e.,* the ability of genes to act as phenotypic capacitors for non-genetically caused phenotypic variability.

## Results

### Haploid fitness and genetic interactions each predict 9% of the variation in phenotypic potential

**Figure 1:**
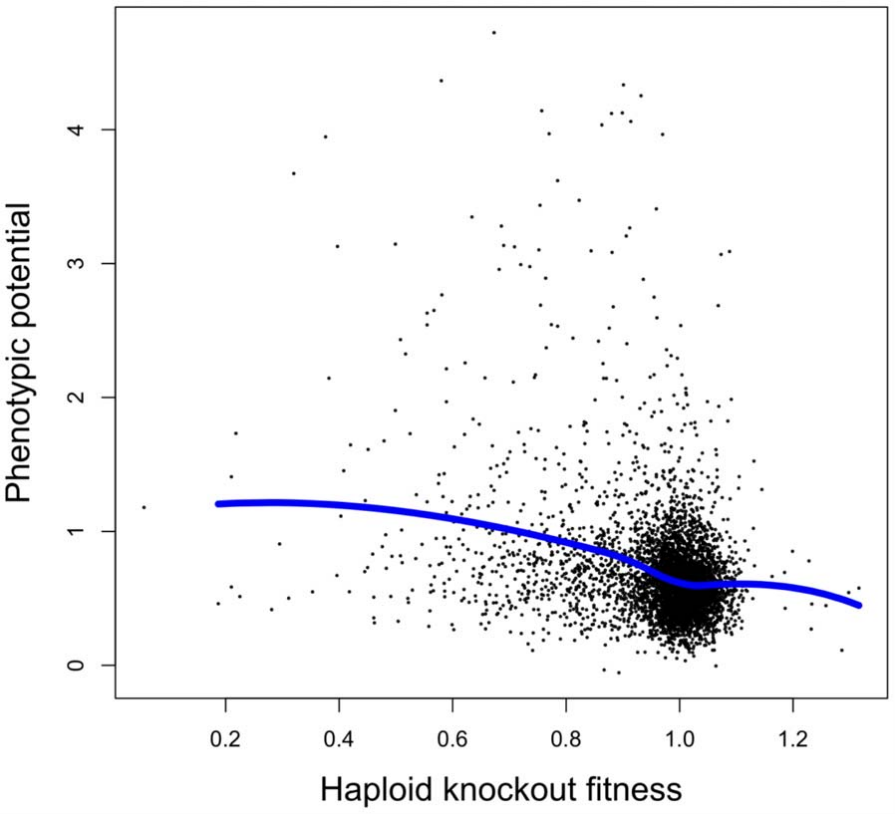
Phenotypic potential of yeast genes (the tendency to induce phenotypic variation in knockouts) is negatively correlated with the genes' haploid knockout fitness (Pearson's *r* = −0.29, *p*<10^−15^). The blue line is a Loess curve fitted to the data.

**Figure 2:**
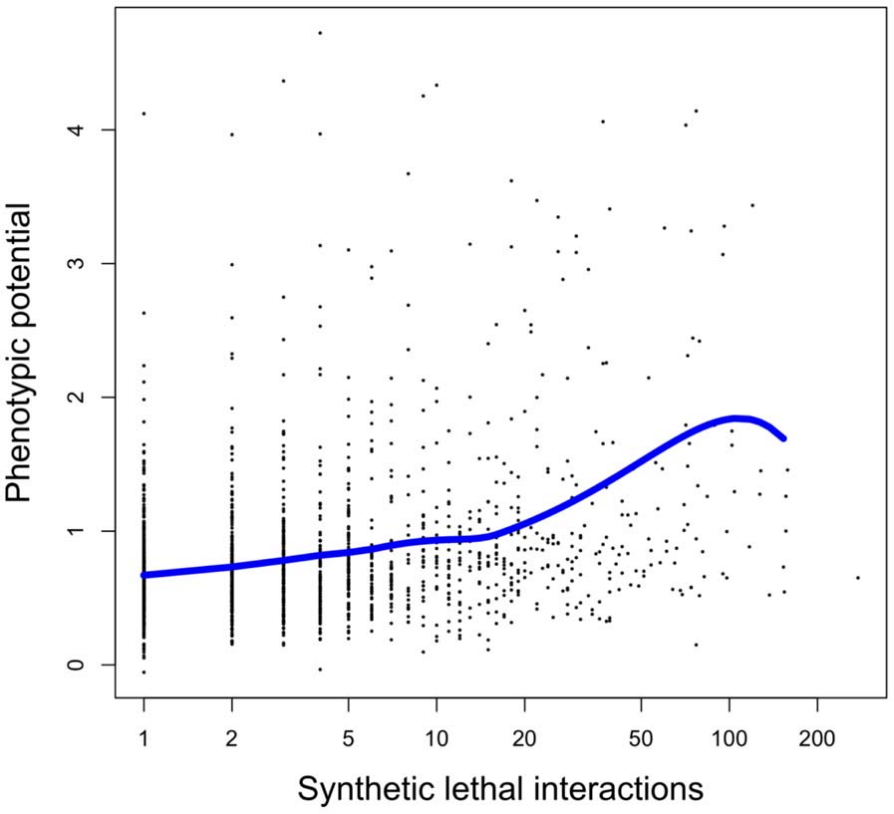
Phenotypic potential of yeast genes (the tendency to induce phenotypic variation in knockouts) is positively correlated with the genes' number of synthetic lethal interactions (Pearson's *r* = 0.30, *p*<10^−15^; interaction number on log-scale). The blue line is a Loess curve fitted to the data.

Based on the arguments outlined above, we expect phenotypic potential to be correlated with the severity of fitness reductions in gene knockouts, and with the number of synthetic lethal interactions. Both predictions were indeed confirmed by experimental data on non-genetically caused morphological variation [9]. Consistent with this earlier result, we find a significant negative correlation between phenotypic potential and haploid fitness (Figure 1; Pearson's *r* = −0.29, *p*<10^−15^), and a significant positive correlation between phenotypic potential and the number of synthetic lethal interactions (Figure 2; *r* = 0.30, *p*<10^−15^; interaction number on log-scale). Thus, when considered individually, each of haploid fitness and connectivity in the synthetic lethal network explain about 9% of the variation in phenotypic potential.

Other variables frequently associated with functional and evolutionary properties of yeast genes are expression level, protein length, and the number of protein-protein interactions. We find that each of these variables shows a weak but statistically significant correlation with phenotypic potential (mRNA expression level: *r* = 0.052, *p* = 0.00011; protein length: *r* = 0.056, *p* = 0.00013; number of protein-protein interactions: *r* = 0.078, *p* = 6.8×10^−5^). As we assess correlations for five different variables, we have to correct for multiple testing here; however, all tests remain statistically significant even after a conservative Bonferroni-correction (corrected *p*<0.0007 in each case).

### Other variables do not add to the predictive power of a combined model

We thus have five variables that are correlated with phenotypic potential – haploid knockout fitness, synthetic lethal interactions, protein length, expression level, and protein-protein interactions. However, it is known that many of these variables are correlated among themselves [17]. Thus, it is possible that only some of these variables are directly connected to phenotypic potential; the effect of other variables might be due to a confounding variable among the former set.

To examine which variables have the strongest explanatory power for variation in phenotypic potential, we employed a linear model, which included haploid knockout fitness, number of synthetic lethal interactions, protein length, mRNA expression level, and number of protein-protein interactions as predictors. This model showed that only haploid fitness, the number of interactions (connectivity) in the synthetic lethal interaction network, and length contribute independently to the variation of phenotypic potential (Table 1); mRNA expression level and protein-protein interaction connectivity do not add any further significant contributions (*p* = 0.62 and *p* = 0.21, respectively). In fact, either haploid fitness or synthetic lethal interactions alone are enough to render the explanatory power of expression level and protein-protein interactions insignificant (*p*>0.18 in each case).

**Table 1:** Statistical significance and percentage explained for the three significant predictor variables for phenotypic potential from a combined linear model.

Predictor	*p*	Percentage explained^1^ (95% CI)
Haploid fitness	<10^−15^	8.8% (6.4%–11.9%)
Synthetic lethal interactions	<10^−15^	4.6% (3.0%–6.8%)
Protein length	0.019	0.3% (0.02%–0.98%)

1 Percent of variation in phenotypic potential explained by each variable independently of the other variables, and 95% confidence intervals (calculated using a relative importance measure that averages over orderings of regressors, with confidence intervals based on 1000 bootstraps [22]).

What is the relative importance of fitness, genetic interactions, and protein length for predicting phenotypic potential? In the combined model, haploid fitness and the number of synthetic lethal interactions explain almost 8.8% and 4.6% of the variation in phenotypic potential, respectively, while the contribution of protein length is minute (Table 1). Thus, as predicted by theory, the extent to which a mutant increases phenotypic variation is indeed correlated to both the fitness effect of the knockout and to the number of severe negative genetic interactions of the mutated protein, while other tested variables appear to be unimportant.

## Discussion

The knockout of a single gene often leads to an increase of phenotypic (*e.g.*, morphological) variability, measured as phenotypic potential in the data analyzed here [1], [9]. Consistent with theoretical expectations, we find that increased phenotypic potential of a gene is associated with stronger (haploid) fitness effects of the knockout mutant, as well as with an increased number of synthetic lethal interactions. Here, we analyze data for non-genetically caused morphological variability. The connection with synthetic lethal interactions thus suggests that phenotypic capacitors indeed often work on both genetically caused and non-genetically caused phenotypic variability, as predicted from theoretical considerations [16].

Levy and Siegal also observed a significant correlation between phenotypic potential and both genetic interactions and haploid growth rate (see their Figure 3E–F, [9]). However, their interpretation differs markedly from ours; they did not consider growth rate an important predictor of phenotypic capacity, stating instead that ‘knockouts of these genes do not tend to cause severe decreases in growth rate’ [9]. This, however, is not surprising: knockout growth rates tend to be bi-modal, with the majority of mutants being either (nearly) lethal or showing little reduction in fitness. As only non-essential genes can be tested for phenotypic potential, severe decreases in growth rate are expected to be rare. However, fitness decreases with a selection coefficient *s* of ‘just’ a few percent are evolutionarily highly relevant in a species with a large effective population size *N*_e_ (with 4*N*_e_ *s* ≫1), as such mutants usually disappear from the population within a few generations [18]. The difference between [9] and the present paper lies largely in the interpretation of the same observations. The previous authors focussed their analysis on network effects; in contrast, we integrated fitness and network effects into a combined model to examine their relative importance.

Based on the function of Hsp90, phenotypic capacitors are often viewed as having direct buffering functions; *i.e.,* it is (often implicitly) assumed that selection has directly acted on the protein's ability to mask genetic or environmental variation [9], [13]. Consistent with theoretical [1] and experimental [8] results, increased variability may instead be a general consequence of functional impairment in complex cellular networks [19]. This may be illustrated by a simple example: if flux through a given metabolic pathway can be maintained by increased production of the pathway substrate, then small variations in the efficiency of pathway enzymes can be compensated. If a mutation in an enzyme that feeds the pathway reduces substrate production capacity, then the previously ‘cryptic’ variation in pathway efficiency will become exposed, resulting in increased phenotypic variability. If a mutation affects the efficiency of important cellular processes, then such ‘domino’ effects may spread far through the network. If this view is correct, then phenotypic potential is not so much a measure of direct buffering, but rather of functional importance and of functional centrality in cellular networks. That phenotypic potential is connected with fitness effects of the single-gene knockout supports this notion; while Hsp90 is a functionally important and central protein, its direct buffering role appears an exception rather than the rule of phenotypic capacitors.

## Methods

### Phenotypic potential

The knockout of individual genes can cause increased phenotypic variability. Here, we used previously published data measuring non-genetically caused morphological variation among the offspring of single-gene deletion *S. cerevisiae* strains [9]. This dataset contains measurements of ‘phenotypic potential’ (i.e., the amount of variation observed across many independent morphological traits in the offspring) for 4683 non-essential genes, resulting in the identification of 502 phenotypic capacitors.

### Other data for *S. cerevisiae*

The number of interactions (connectivity) of proteins in the protein-protein interaction network and in the synthetic lethal network was obtained from [9]. mRNA expression level was obtained from a genome-wide microarray analysis [20]. Haploid fitness was obtained from [21].
